# Biodegradable curcumin-nanoclay films for extending shrimp shelf-life and freshness

**DOI:** 10.1016/j.crfs.2025.101102

**Published:** 2025-05-29

**Authors:** Kalpani Y. Perera, Sneha Sabu Mathew, Luana de S.C. Carnaval, Dileswar Pradhan, Amit K. Jaiswal, Swarna Jaiswal

**Affiliations:** a School of Food Science and Environmental Health, Faculty of Sciences and Health, Technological University Dublin-City Campus, Central Quad, Grangegorman, Dublin, D07 ADY7, Ireland; b Centre for Sustainable Packaging and Bioproducts (CSPB), Technological University Dublin-City Campus, Central Quad, Grangegorman, Dublin, D07 ADY7, Ireland; c Sustainable and Health Research Hub (SHRH), Technological University Dublin-City Campus, Grangegorman, Dublin, D07 H6K8, Ireland; d Health Engineering & Materials Science Research Hub, Technological University Dublin - City Campus, Grangegorman, Dublin, Ireland

**Keywords:** Curcumin, Nanoclay, Biodegradable packaging, Intelligent films, Shrimp shelf-life

## Abstract

Preserving food freshness is fundamental for minimizing waste. Intelligent packaging offers valuable information, enhances safety, and improves food quality during storage and transit. This study aimed to develop biodegradable, intelligent food packaging for shrimp using curcumin (Cur), gelatin (Gel), sodium alginate (SA), and nanoclay (NC) to enhance food safety and extend shelf life. The films with 1 % NC-Cur exhibited a 3.46-fold increase in UV barrier properties compared to the control, although Cur reduced transparency. Mechanical testing showed that films containing 0.5 % NC-Cur achieved optimal tensile strength (38.6 ± 30.9 MPa). The water contact angle (WCA) increased to 84.12 ± 3.14°, indicating enhanced hydrophobicity, while solubility decreased significantly. The films demonstrated pH-responsive color changes (yellow to brick red) and improved antioxidant activity, with 1 % NC-Cur scavenging 35.55 % radicals compared to 15.10 % in the control. Biodegradation studies confirmed complete degradation within 30 days, although films with higher NC levels showed slower curcumin migration. After six days, shrimp stored at 4 °C exhibited a yellow-to-orange color change due to microbial growth and pH fluctuations. Packaging with NC-Cur extended shrimp shelf life by two days. These findings demonstrate the potential of NC, Cur, Gel, and SA-based films for intelligent, eco-friendly packaging that enhances food quality, delays spoilage, and reduces environmental impact.

## Highlights

• Biodegradable films developed with curcumin, gelatin, sodium alginate, and nanoclay.
• Enhanced mechanical, UV barrier, and antioxidant properties of packaging films.
• Films exhibit pH sensitivity, enabling real-time shrimp freshness monitoring.
• Curcumin's controlled release extends shrimp shelf life by two days.
• Fully biodegradable films (within 30 days) promote sustainable food packaging solutions.

## Graphical abstract

**Figure:**
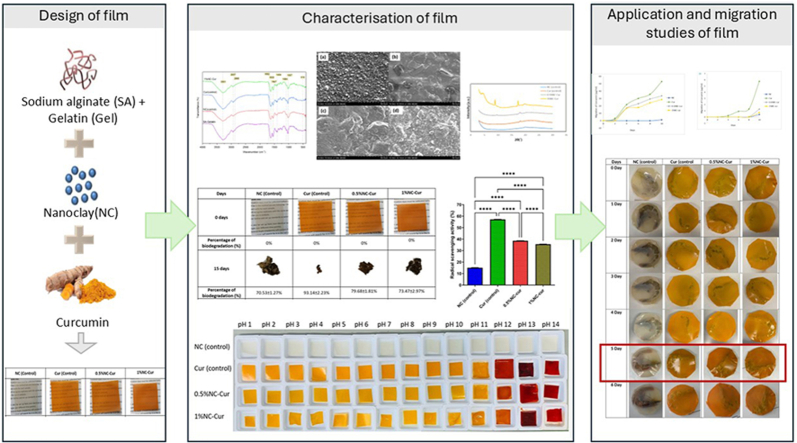

## 1 Introduction

Food waste represents a significant social, environmental, and economic challenge worldwide (Etxabide et al., 2021a). In the European Union, nearly 40 % of the total 129.2 million tons of food waste is generated at the household level, with 20 %–25 % of this waste attributed to packaging inefficiencies (Etxabide et al., 2021a). A primary factor contributing to these inefficiencies is the failure of traditional food packaging to indicate freshness or deterioration, resulting in the unwarranted disposal of still-consumable food (Etxabide et al., 2021a).

As consumer awareness of food safety and quality increases with improving living conditions, the demand for packaging that guarantees and conveys freshness has escalated. Consequently, monitoring and maintaining food freshness remains a significant challenge in the food industry (Wang et al., 2022b). Intelligent or smart packaging systems, designed to provide information, enhance food safety, and maintain quality during storage and distribution, have garnered significant interest (Liu et al., 2018). These packaging systems often include chemical indicators capable of detecting the presence or concentration of specific compounds and producing a visible signal (Etxabide et al., 2021a). For example, some indicators detect volatile organic compounds (VOCs) such as acetic acid and amines, released during spoilage (Yildiz et al., 2021). These VOCs cause pH changes that trigger a color shift in the packaging film, providing consumers with an immediate visual cue about food quality (Etxabide et al., 2021a).

Due to the increasing demand for safe and sustainable solutions, the incorporation of food-grade, non-toxic colourants in these intelligent systems has become imperative. Natural dyes, particularly those sourced from plants, provide an eco-friendly substitute for synthetic indicators (Etxabide et al., 2021a).

In parallel, recent research has increasingly focused on bioactive packaging films with antibacterial and antioxidant properties, as these films help preserve food safety and extend shelf life by inhibiting microbial growth and oxidative degradation (Wang et al., 2022b). Antioxidants embedded in the films may migrate to the food, reducing the need for chemical additives (Ma et al., 2017).

This is especially pertinent for extremely perishable products like seafood. For example shrimp, is prone to spoilage through autolysis, lipid and protein oxidation, biogenic amine formation, and microbial contamination. Thus, adequate seafood packaging must inhibit oxidation, prevent microbial growth, and provide thermal insulation (Laorenza et al., 2022). Currently, chemical freshness indicators are used for seafood, with colourimetric films gaining popularity due to their ability to detect pH changes caused by the release of volatile amines during spoilage (Chen et al., 2020). Among the natural dyes investigated for this purpose, curcumin stands out by its multifunctional characteristics.

Curcumin, a bioactive compound derived from turmeric, has well-known anti-inflammatory and antioxidant properties. Widely used as a natural food colorant, curcumin's pH-dependent solubility makes it an ideal indicator for intelligent packaging (Chen et al., 2020). Under acidic conditions, curcumin-based films appear yellow, shifting to orange or red as alkalinity increases due to the formation of phenoxide anions. However, its application is limited by its sensitivity to light, oxygen, and temperature, necessitating strategies to improve its stability in packaging materials (Liu et al., 2022).

Despite recent studies incorporating curcumin in packaging films, research focusing specifically on shrimp preservation remains limited. Previous studies have incorporated curcumin with soy protein isolate-cellulose nanocrystals (Xiao et al., 2021), soy protein isolate (Liu et al., 2022), agar-polyvinyl alcohol (Zhang et al., 2021), and poly(lactic acid)-poly(propylene carbonate) (Cvek et al., 2022). However, improvements in performance through combining nanoparticles and polysaccharide-based films have been marginal (Roy & Rhim, 2020). The above combinations often fall short in mechanical strength or functional effectiveness, highlighting the need for more robust formulations.

To address this gap, the present study combined gelatin and sodium alginate (SA) biopolymers with curcumin and nanoclay (NC) to develop food packaging materials. Gelatin is a water-soluble protein with strong UV absorption properties and antioxidant capacity. However, gelatin-based films are prone to moisture absorption and exhibit poor tensile strength (Chaari et al., 2022). To address these limitations, gelatin is often blended with lipids, polysaccharides, and polymers to improve extensibility and mechanical properties. SA, an inexpensive and biocompatible polysaccharide, is widely used in biodegradable food packaging due to its transparency, film-forming ability, and mechanical strength (Perera et al., 2023). The addition of nanoclay enhances the barrier, mechanical, and thermal properties of biopolymer-based films (Iamareerat et al., 2018), as the clay layers act as barriers to gas and vapour permeability, thereby improving food preservation (Hosseini et al., 2020).

This study aims to develop and evaluate gelatin-SA-curcumin-nanoclay films for shrimp packaging. By leveraging the synergistic effects of curcumin and nanoclay, the films are designed to extend shrimp shelf life by inhibiting microbial growth and offering visual freshness indicators. This approach enhances food preservation and aligns with sustainability goals, making the films an environmentally friendly packaging solution.

## 2 Materials and methods

### 2.1 Materials

SA (alginic acid sodium salt from brown algae), nanoclay-hydrophilic bentonite (also known as Montmorillonite clay, bentonite, Nanomer®PGV, and Nanomer®clay) (NC) formula: H_2_Al_2_O_6_Si, the average particle size of <25 μm and TWEEN® 80 were obtained from Sigma Aldrich (Ireland). Gelatin, and glycerol solution were purchased from Thermo Fisher Scientific, Ireland. 95.01 % Curcumin turmeric extract was purchased from a local health shop in Ireland.

### 2.2 Development of intelligent food packaging films

Various combinations of films, including control films, were prepared for this study. Different concentrations of nanoclay (NC) were added to 100 mL of distilled water to achieve final concentrations ranging from 0.3 % (w/v) to 2 % (w/v). Based on preliminary assessments of key food packaging characteristics—including appearance, mechanical properties, and water contact angle, the two most effective concentrations, 0.5 % (w/v) and 1 % (w/v) NC, were selected. Additionally, 0.3 % (w/v) curcumin (Cur) was incorporated into all films except for the NC control film, as this concentration was identified as optimal for pH colour change based on prior studies. The resulting films were designated as NC (control), Cur (control), 0.5 % NC-Cur, and 1 % NC-Cur.

The prepared solutions were stirred for 2 h at room temperature at 1500 rpm. Subsequently, the mixtures underwent ultrasonic processing using an ultrasonic processor (130 W, 20 kHz) for 15 min. To enhance flexibility, 0. 5 % (v/v) glycerol was added as a plasticiser, followed by 1 g of sodium alginate (1 % w/v), stirred at 60 °C and 600 rpm for 1 h until completely dissolved. Next, 2 g of gelatin (2 % w/v) was added, and the solution was stirred for 30 min at 60 °C and 400 rpm until fully dissolved.

The prepared solutions were poured into square plastic petri dishes and air-dried at room temperature for 48 h. Once the films dried, they were carefully removed from the petri dishes. Prior to testing, the films were conditioned for at least 48 h at 50 % relative humidity (RH) and 25 °C. All film characterisations and tests were performed in triplicate for statistical accuracy.

### 2.3 Characterization of the intelligent food packaging films

#### 2.3.1 Light transmittance, UV barrier property, and surface color

The colour properties of the films, including L∗ (brightness), a∗ (red-green), and b∗ (yellow-blue), were measured using a ColorQuest XE spectrophotometer (Hunter Lab). Colour measurements were carried out under standard viewing conditions using Illuminant D65 and a 10° standard observer. A standard white calibration plate with the parameters L∗ = 97.75, a∗ = −0.42, and b∗ = 1.85 was used as a reference. Six readings were taken from different locations on each film sample to determine the Hunter colour values (L∗, a∗, and b∗). The films' total colour difference (ΔE) was calculated using Eqn. (1):

(1) ΔE = [(ΔL)^2^+(Δa)^2^+(Δb)^2^]^0.5^

where ΔL, Δa, and Δb respectively represent the differences between values of the white colour plate and prepared film (Sharma et al., 2020).

To assess the films' UV-light barrier properties and transparency, an Agilent UV–Vis spectrophotometer (Agilent Technologies, Cork, Ireland) was used. The percentage transmittance at 280 nm (T280) and 660 nm (T660) was measured. Rectangular film samples (3 cm × 7 cm) were prepared and mounted between the spectrophotometer's magnetic cells for analysis.

#### 2.3.2 Chemical structural properties

The chemical structural properties of the intelligent packaging films were analysed using attenuated total reflectance-Fourier transform infrared (ATR-FTIR) spectroscopy (Thermo Scientific, Ireland). This technique was employed to identify the functional groups present in the films. The measurements were conducted at a resolution of 4 cm^−1^, covering a wavenumber range of 4000–500 cm^−1^. Film samples measuring 4 × 4 cm were placed directly onto the ATR stage for spectral analysis.

#### 2.3.3 Surface morphological properties

The films' surface morphological properties were examined using a Hitachi SU-70 scanning electron microscope (SEM, USA). To enhance conductivity, a 6 nm layer of Au/Pd coating was applied to the film samples using a sputter coating machine. The coated samples were mounted onto the SEM sample holder for inspection. Images of the film surfaces were captured at 10,000 × magnification with an operating voltage of 10 kV.

#### 2.3.4 Thickness and mechanical properties

The thickness of the film samples was measured using a digital micrometer (VWR, Ireland) with an accuracy of 0.001 mm. Measurements were taken at 12 random points across the surface of each film sample.

The mechanical strength of the packaging films is crucial for ensuring food protection during storage, handling, and processing. The tensile properties were evaluated following ASTM D 882–88 standards using an Instron Universal Testing Machine (Model 5565, Instron Engineering Corporation, Canton, MA, USA). The nanocomposite films were cut into rectangular strips measuring 3 × 15 cm. The tests were conducted at room temperature with a 50 mm grip length, a crosshead speed of 50 mm/min, and a 500 N load cell until the film samples broke.

Tensile strength (TS), elongation at break (EB), and elastic modulus (EM) were assessed to determine the films' flexibility and mechanical strength. The TS (MPa) and EB ( %) were calculated using the following equations:

(2) $$\text{TS}=\frac{F}{X*w}$$

(3) $$\text{EB}=\frac{L_{f}-L_{0}}{L_{0}}\times 100$$

Here, x (mm) is the sample thickness, W (mm) is the sample width, L_f_ is the film elongation length at the break, and L_o_ (50 mm) is the initial grasping length of the film. The elastic modulus (GPa) represents the film's resistance to elastic deformation. It is determined from the slope of the stress-strain curve within the elastic region, defined as the ratio of stress to strain.

#### 2.3.5 X-ray diffraction (XRD)

The crystallinity of the nanoclay films was analysed using X-ray diffraction (XRD) with a Rigaku Miniflex benchtop X-ray diffractometer equipped with a Cu target and Kα X-ray source. The wavelengths for the X-ray emissions were Kα1 = 1.54059 Å, Kα2 = 1.54441 Å, and Kβ = 1.39225 Å, with a Kα12 ratio of 0.4970 and horizontal polarization of 0.500.

The XRD analysis was performed with the tube voltage set at 40 kV and a tube current of 15 mA. The scan was conducted using a θ/2θ scan axis and a 2D scanning method. The scanning range was set from 3° to 30°, with a step size of 0.01° and a scan speed of 3° per minute.

#### 2.3.6 Water contact angle (WCA)

The water contact angle (WCA) of the film surfaces was measured using a dynamic contact angle analyser (FTA-200 system) to evaluate the surface's hydrophobicity or hydrophilicity. Rectangular film samples (3 × 8 cm) were placed on a stainless-steel platform equipped with the contact angle analyser.

A micro syringe dispensed approximately 10 μL of distilled water onto the film surface. A high-speed camera recorded the interaction between the water droplet and the film surface. The captured image was processed using computer software to determine the contact angle.

#### 2.3.7 Water vapour permeability

The films' water vapour permeability rate (WVPR) was determined gravimetrically following the method described by Salama et al. (2018). Circular containers with a diameter of 30 mm were filled with 15 g of oven-dried calcium chloride (CaCl_2_). The tested films (n = 3) were used to seal the tops of the containers, while unsealed containers served as controls.

The containers were placed in a chamber maintained at 25 °C and 100 % relative humidity for four days. The weight of each container was recorded every 12 h. The WVPR (g·m^−2^·h^−1^) was calculated using Equation (4):

(4) $$\text{WVPR}=\frac{W}{\text{AXt}}$$

Where, "W" represents the weight loss of the sample, "A" represents the film's surface area, and "t" represents the period in hours.

#### 2.3.8 Water solubility and moisture content

To determine the water solubility of the films, rectangular samples (3 × 1 cm) were prepared in triplicate and dried in an oven at 105 °C for 24 h to obtain the initial dry weight (*Wi*, in grams). Each sample was then submerged in 50 mL of distilled water in a beaker and stirred magnetically at 500 rpm for 6 h at room temperature (23 ± 2 °C). After immersion, the insoluble portion of the film was removed, dried in an oven at 105 °C for 24 h, and reweighed to obtain the final weight (*W*_*f*_, in grams). The percentage of weight loss, indicating water solubility, was calculated using Equation (5):

(5) Weight loss % = [(W_i_-W_f_)/ W_i_] ∗ 100

Here, “W_i_” is the initial weight and “W_f_” is the final weight.

The moisture content of the films was determined based on the method described by Shanbhag et al. (2023), following the ASTM D4442 standard. Square film samples (2.0 cm^2^) were initially weighed (*W1*, in grams) and then dried in an oven at 100 °C until a constant weight was achieved. The final weight (*W2*, in grams) was recorded. The percentage of moisture content was calculated using Equation (6):

(6) $$\text{Moisture}\;\text{content}\;\left(\;\%\right)=\frac{\left(W1-W2\right)}{W2}\times 100$$

### 2.4 Antimicrobial activity

The antimicrobial properties of the intelligent packaging films were evaluated using the Japanese Industrial Standard (JIS Z 2801:2000) method against foodborne pathogenic bacteria: *Staphylococcus aureus* (ATCC 25923) (Gram-positive) and *Escherichia coli* (ATCC 25922) (Gram-negative). The testing was conducted at 0 and 24 h using 5 × 5 cm^2^ film samples. The bacterial inoculum was prepared with an initial concentration of 10^6^ CFU/mL using the McFarland standard. For testing, a filter paper moistened with sterilized water was placed in a Petri dish, followed by a 5.5 × 5.5 cm^2^ glass slide placed on swab sticks positioned in the dish. The glass slides were coated with 400 μL of the test inoculum and covered with the packaging films. The setup was incubated at 37 °C for 24 h, while 0-h samples were tested immediately using the spread plate method. To assess antimicrobial activity, the film samples were placed in sterilized stomacher bags with 10 mL of Maximum Recovery Diluent (MRD) and mixed in a stomacher (AGB Scientific-Lab Blender 400) for 40–45 s. Viable bacterial counts were determined by serial dilution of the MRD culture and plating on nutrient agar plates.

### 2.5 pH sensitivity studies and antioxidant activity

The pH sensitivity of the intelligent packaging films was tested using a range of pH solutions from 1 to 14. The films' colour changes were visually observed across the pH spectrum.

To evaluate the antioxidant activity, the DPPH assay was performed following the method described by Maryam Adilah et al. (2018). For the extract preparation, 25 mg of the film sample was submerged in 3 mL of ethanol. Subsequently, 3 mL of the film extract was mixed with 1 mL of ethanolic DPPH (0.1 mM) solution. The mixture was thoroughly mixed and incubated in a dark chamber at room temperature for 30 min. The absorbance of the solution was measured at 517 nm using a spectrophotometer.

The DPPH radical scavenging activity ( %) was calculated using Equation (7):

(7) $$\text{Radical}\;\text{scavenging}\;\text{activity}\;\left(\;\%\right)=\frac{\left(\text{Absorbance}\;\text{of}\;\text{DPPH}-\text{Absorbance}\;\text{of}\;\text{sample}\right)\times 100}{\text{Absorbance}\;\text{of}\;\text{DPPH}}$$

### 2.6 Migration of curcumin

The migration of curcumin (Cur) was assessed in two food simulants: olive oil (simulant D) and 3 % (v/v) aqueous acetic acid (simulant B). The procedure followed the British Standard EN1186–1:2002 for "materials and articles in contact with food stuffs," with minor modifications to the protocol described by Schaefer et al. (2020).

Film samples (3 × 5 cm) were immersed in 50 mL of the simulants (olive oil and 3 % acetic acid) and incubated at 25 °C for 10 days. The films were removed at different time points (0, 2, 4, 6, 8, and 10 days). The concentration of curcumin released into the simulants was measured by recording the absorbance at a wavelength of 425 nm using a UV–Vis spectrophotometer. The amount of curcumin migrated was quantified using a curcumin standard curve.

### 2.7 Biodegradability studies of the intelligent food packaging films

The biodegradability of the formulated films was assessed according to ASTM Standard D5988–18, "Standard Test Method for Determining Aerobic Biodegradation of Plastic Materials in Soil." The biodegradation process was monitored by measuring CO_2_ production, expressed as a percentage of the material's carbon content over time, to determine the extent of biodegradation.

The experiment used a soil-contact incubation apparatus containing 200 g of soil and 50 mL of 0.5 N potassium hydroxide (KOH) in a beaker. The setup included technical controls, soil blanks, and known-weight film samples tested in triplicate. The apparatus was maintained at 25 ± 2 °C and 95 % relative humidity. Biodegradation measurements were taken at 0, 15, and 30, day intervals.

The amount of carbon dioxide produced at each time point was quantified by titrating the KOH solution with 0.05 N hydrochloric acid (HCl) to a phenolphthalein endpoint. The percentage of biodegradation was calculated using Equation (8):

(8) $$\text{Percentage}\;\text{of}\;\text{biodegradation}=\frac{\text{Weight}\;\text{of}\;\text{carbondioxide}\;\text{produced}}{\text{Weight}\;\text{of}\;\text{theoritical}\;\text{carbondioxide}}\times 100$$

### 2.8 Application of films for indicating the freshness of shrimp

The application study was conducted by wrapping shrimp samples with the designed food packaging films: NC (control), Cur (control), 0.5 % NC-Cur, and 1 % NC-Cur. Shrimp samples were stored at 4 °C for up to 14 days to monitor spoilage progression. Observations were conducted daily for the first 6 days. At each time point, pH, total volatile basic nitrogen (TVB-N), and visual appearance were assessed. Total bacterial count (TBC) and film colour were measured every other day throughout the 14-day storage period. The TVB-N content was determined using the method described by Mohammadalinejhad et al. (2020).

For the TVB-N analysis, 10 g of shrimp were mixed with 300 mL of distilled water and homogenized. The homogenized sample was transferred to a Kjeldahl flask, and 2 g of magnesium oxide (MgO) was added. The mixture was heated until it reached boiling, after which the apparatus was sealed. The steam distillate was collected in a flask containing 25 mL of 2 % boric acid mixed with methylene blue and methyl red indicators. The distillation process continued for 25 min.

The resulting solution was titrated with 0.1 N sulfuric acid until the colour changed from green to dark pink, indicating the titration endpoint. The TVB-N content (mg N/100 g) was calculated using Equation (8):

TVB-N content = *V*_*1*_ (ml) × 14 (8)

where *V*_*1*_ is the volume (mL) of sulfuric acid used during titration. The visual appearance and the colorimetric value of all the films were also determined at each time point.

### 2.9 Statistical analysis

The statistical analysis was performed using Statistics Centurion XV software (Stat Point Technologies Inc., Warrenton, VA, USA). Analysis of variance (ANOVA) was conducted, followed by Fisher's least significant difference (LSD) test for multiple comparisons. Bar graphs were created using GraphPad Prism (version 9.1.0) and Origin 2023 software. All values were reported as the mean ± standard deviation (SD), with a significance level set at 5 % (*p* < 0.05).

## 3 Results and discussion

### 3.1 Characterization of the intelligent food packaging films

#### 3.1.1 Light transmittance, UV barrier properties, and surface colour

The surface colour, light transmittance, and UV barrier properties of the bio-nanocomposite films are summarized in Table 1. Colour is a critical parameter for food packaging as it directly affects the appearance and consumer perception of the product (Etxabide et al., 2021a). In the developed films, the lightness (*L* value) decreased with the addition of curcumin (Cur) and the combination of Cur and nanoclay (NC). Specifically, the *L* value of the 1 % NC-Cur film was 1.28 times lower than that of the NC control, indicating a darker film.

**Table 1:** Surface colour, Light transmittance, and UV barrier properties of active packaging films.

Films	L (Lightness)	a (Red-Green)	b (Yellow-Blue)	ΔE	Transmittance T (280 nm) UV barrier property ( %)	Transmittance T (600 nm) film transparency ( %)
NC (control)	83.57 ± 0.29^d^	(−)0.17 ± 0.05^a^	10.61 ± 0.24^a^	84.24 ± 0.26^a^	0.09 ± 0.008^a^	16.01 ± 0.29^d^
Cur (control)	67.15 ± 0.28^c^	29.54 ± 0.03^d^	64.09 ± 0.34^d^	97.42 ± 0.31^d^	0.38 ± 0.08^b^	3.59 ± 0.61^c^
0.5 %NC-Cur	64.21 ± 0.37^a^	28.82 ± 0.29^c^	59.77 ± 0.77^b^	92.34 ± 0.79^b^	0.09 ± 0.01^a^	2.25 ± 0.18^b^
1 %NC-Cur	65.72 ± 0.31^b^	26.99 ± 0.36^b^	61.8 ± 0.37^c^	94.16 ± 0.42^c^	0.11 ± 0.04^a^	1.72 ± 0.09^a^

∗The letters (a–d) indicate groups that are significantly different (p < 0.05).

The Cur control film exhibited the highest yellowness (*b* value), but this was reduced with the addition of NC, likely due to the dispersion of Cur within the NC matrix. Compared to the NC control, the *b* value increased by 5.82 times in the 1 % NC-Cur film. Including active compounds like Cur and NC structurally alters the film matrix, thereby influencing its colour, as Ma et al. (2017) noted. Films without Cur exhibited the highest *L* (96.21) and the lowest *a* (1.18) and *b* (0.41) values, consistent with the findings of Li et al. (2022).

UV barrier properties are essential in food packaging to prevent quality deterioration caused by light, such as vitamin degradation, discolouration, and fat oxidation (Etxabide et al., 2021a). The addition of Cur significantly enhanced the UV barrier properties, with a 3.46-fold improvement observed in the 1 % NC-Cur film compared to the NC control. The Cur control film exhibited the highest UV barrier performance, with minimal UV transmittance (0.09 ± 0.01^a^ %). Similarly, the 1 % NC-Cur film demonstrated excellent UV blocking capacity (0.11 ± 0.04^a^ %), consistent with the findings of Roy and Rhim (2020). These results suggest that Cur's high UV absorption ability contributes significantly to the films' protective properties.

Transparency, assessed by light transmittance at 600 nm, decreased with the addition of Cur. The transparency of the 1 % NC-Cur film (1.72 ± 0.09 %) was reduced by 9.3 times compared to the NC control (16.01 ± 0.29 %). This reduction aligns with prior studies showing that colorants like Cur decrease transparency by scattering and absorbing light within the film matrix (Etxabide et al., 2021a). For instance, Cur incorporation reduced the transparency of carrageenan films from 91.29 % to 18.25 %, as reported by Liu et al. (2018). Similarly, as Cur concentration increased, transmittance decreased from 49.43 % to 17.86 %, a trend attributed to Cur's strong UV absorption properties (Liu et al., 2022).

Moreover, the synergistic effects of Cur and NC were evident in the enhanced UV barrier and reduced transparency of the 1 % NC-Cur film. These findings are consistent with Roy and Rhim (2020), who demonstrated that the addition of Cur and ZnO to carboxymethyl cellulose films improved UV shielding but reduced transparency. The combined incorporation of Cur and NC in this study effectively shielded packaged food from visible light and UV radiation, highlighting their suitability for intelligent food packaging applications.

#### 3.1.2 Chemical structural properties

**Fig. 1:**
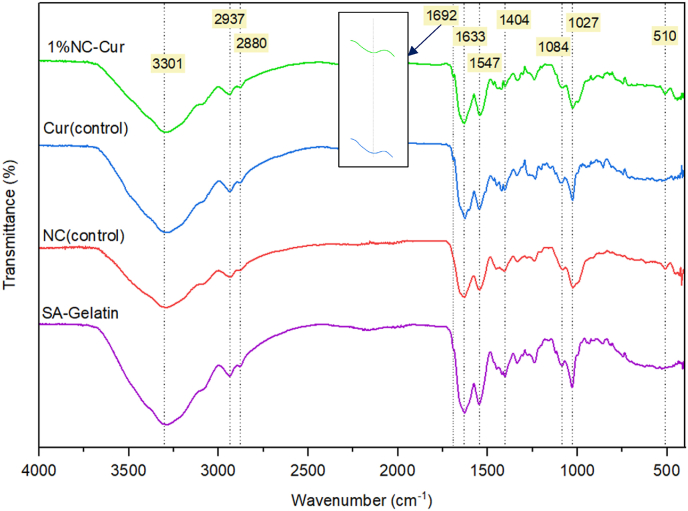
FTIR spectrum of SA-Gel (control), NC (control), Cur (control), and 1 % NC-Cur intelligent food packaging films.

FTIR is commonly used to assess the miscibility and compatibility of biopolymers and nano-fillers due to its rapid and non-destructive nature. Changes in chemical functional groups can be identified through shifts in absorption bands in the FTIR spectrum, as illustrated in Fig. 1 (Roy and Rhim, 2020). The FTIR results in this study revealed biocompatibility between sodium alginate (SA) and gelatin, as evidenced by the presence of characteristic functional groups associated with both biopolymers. The observed interactions between SA and gelatin were mediated by saturated and unsaturated linkages, such as C–O stretching at 1027 cm^−1^, C=O carbonyl groups, and C ≡ C alkyne groups at 1547 cm^−1^. Gelatin specifically exhibited N–H stretching of amide II and C–N stretching at 1549 cm^−1^ (Roy and Rhim, 2021).

Upon the addition of nanoclay (NC), minor peaks were observed in the fingerprint region, with a significant absorption band at 510 cm^−1^ corresponding to the Al–O–Si bonds of NC (Mohammad et al., 2021). The incorporation of curcumin (Cur) was confirmed by distinct peaks at 1692 cm^−1^, indicative of free C–H vibration. Similar studies have reported additional peaks for Cur, such as at 3504 cm^−1^ and 1626 cm^−1^, attributed to the free vibration of O–H and C–H groups, respectively, while peaks at 1602 cm^−1^ and 1509 cm^−1^ were primarily linked to C–C stretching in the benzene ring (Liu et al., 2022). Peaks at 1633 cm^−1^, detected in this study, were attributed to the carbonyl groups of Cur (Roy and Rhim, 2020). Furthermore, aromatic C–O and C–O–C stretching was observed at 1404 cm^−1^, 1084 cm^−1^, and 1027 cm^−1^, consistent with findings by Li et al. (2022).

The variations in the intensity and position of the FTIR peaks in the bio-nanocomposite films suggest non-covalent interactions, such as van der Waals forces and hydrogen bonding, between the filler (NC) and the biopolymer matrix. Roy and Rhim (2020) previously noted that these interactions can modulate the structural compatibility and stability of such composite films. Based on the FTIR results, we hypothesise that the observed minor variations are attributable to changes in non-covalent interactions between the biopolymer matrix, the active component (Cur), and the nano-filler (NC), contributing to the films' structural integrity and functional performance.

#### 3.1.3 Surface morphological properties

**Fig. 2:**
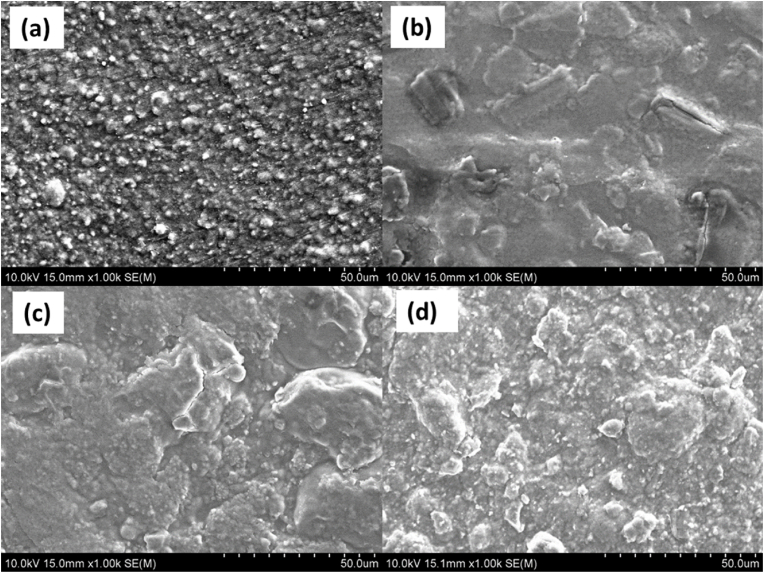
SEM surface images, 10 kV × 15 mm x 1k of intelligent food packaging materials (a) NC (control), (b) Cur (control), (c) 0.5 %NC-Cur and (d) 1 %NC-Cur.

The surface morphology of the food packaging films was analysed using scanning electron microscopy (SEM) under parameters of 10 kV, a 15 mm working distance, and 1000 × magnification, as shown in Fig. 2. The SEM images revealed distinct morphological changes in the films depending on the inclusion of nanoclay (NC) and curcumin (Cur).

As observed in the NC control film (Fig. 2a), the addition of NC to the polymer matrix increased surface roughness, consistent with its particulate nature. In films with Cur alone (Fig. 2b), visible lumps were observed on the surface, indicating the presence of Cur particles that were not uniformly distributed throughout the matrix. However, in films containing both NC and Cur (Fig. 2c and d), the surface roughness decreased, and the Cur lumps were evenly dispersed across the surface. This uniform distribution suggests that Cur and NC were effectively incorporated and well-blended into the polymer matrix.

Previous studies support these findings, demonstrating that rod-shaped Cur crystals were evenly distributed in a carrageenan biopolymer matrix, which improved the mechanical and barrier properties of the films (Liu et al., 2021). However, it has also been reported that increasing the Cur content can deteriorate film homogeneity, resulting in visible aggregation. At higher concentrations, Cur crystals may precipitate and randomly stack within the matrix as the solvent evaporates during the drying process (Liu et al., 2021).

Similarly, Li et al. (2022) reported that Cur significantly impacts the morphology and structure of the film matrix, with higher Cur concentrations leading to rougher and more cracked surfaces. In the present study, the Cur concentration was kept low, preventing the formation of cracks and ensuring a smooth surface. Aggregates that formed may be attributed to the dispersion of Cur within the matrix, which can create irregular micron-sized shapes, as described by Xu et al. (2021). Roy and Rhim (2020) also noted that such aggregation occurs during the solvent evaporation stage, contributing to surface irregularities.

The SEM analysis in this study indicates that the incorporation of NC and a controlled concentration of Cur resulted in films with improved morphological uniformity, which is essential for enhancing their mechanical and functional properties.

#### 3.1.4 Thickness, and mechanical properties

The thickness and mechanical properties of the intelligent packaging films are summarized in Table 2. The thickness of all films was around 0.1 ± 0.0 mm, with no significant differences observed after the addition of curcumin (Cur). A consistent thickness is critical for maintaining the uniformity and performance of packaging films. For effective storage and transport, intelligent active films must possess mechanical resistance and flexibility (Wang et al., 2022b).

**Table 2:** Thickness and the mechanical properties food packaging films.

Films	Thickness (mm)	Tensile Strength TS (MPa)	Elongation at Break EB ( %)	Elastic modulus EM (MPa)
NC (control)	0.102 ± 0.001^a^	38.15 ± 1.54^c^	2.65 ± 1.31^a,b^	0.72 ± 0.34^b^
Cur (control)	0.103 ± 0.002^a^	3.29 ± 1.13^a^	0.63 ± 0.75^a^	0.18 ± 0.10^a^
0.5 %NC-Cur	0.101 ± 0.001^a^	38.63 ± 0.93^c^	3.89 ± 0.69^b^	0.78 ± 0.08^b^
1 %NC-Cur	0.103 ± 0.001^a^	16.02 ± 2.43^b^	3.06 ± 1.28^b^	0.85 ± 0.05^b^

∗The letters (a–d) indicate groups that are significantly different (p < 0.05).

Tensile strength (TS) measures the maximum stress a film can withstand before breaking, while elongation at break (EB) reflects the film's ability to stretch without failure (Ma et al., 2017). Incorporating nanoparticles (NPs) or active substances into biopolymer-based films often improves mechanical properties by enhancing the polymer matrix.

The Cur control film exhibited the lowest TS (3.29 ± 1.13^a^ MPa). The highest TS was observed in the 0.5 % NC-Cur film at 38.6 ± 30.9^c^ MPa, an 11.74-fold increase compared to the Cur control. This improvement is attributed to the moderate addition of NC, which reinforces the polymer structure. However, increasing the NC and Cur content in the 1 % NC-Cur film led to a decrease in TS (16.02 ± 2.40^b^ MPa), likely due to excess additives disrupting the uniformity of the polymer matrix. Similar trends have been reported; for instance, Fathi et al. (2022) found that adding Cur to a pectin-polyvinyl alcohol matrix reduced TS from 41.65 MPa to 34.82 MPa. Similarly, Ma et al. (2017) reported a reduction in TS from 44.22 MPa to 25.10 MPa in tara gum-polyvinyl alcohol films containing 0.5 % Cur, due to interactions between Cur's phenolic hydroxyl groups and the matrix.

Contrasting findings by Li et al. (2022) suggest that in some matrices, TS increases with higher Cur content, such as in Konjac glucomannan films, where TS improved from 29.26 MPa to 52.37 MPa as Cur content increased from 0 % to 3 %. These discrepancies likely stem from differences in polymer compatibility and the interactions between Cur and specific biopolymer matrices.

The Cur control film also had the lowest EB, indicating poor flexibility. Incorporating NC significantly enhanced EB, with the 0.5 % NC-Cur film exhibiting the highest EB (3.89 ± 0.69^b^ %), a 6.17-fold increase compared to the Cur control. The combination of NC and Cur improved the polymer matrix's elasticity, consistent with findings by Fathi et al. (2022), who observed that adding Cur increased EB in similar packaging films. The improved EB may result from weakened intermolecular interactions caused by Cur, allowing for greater film flexibility (Ma et al., 2017).

Interestingly, while Cur enhanced EB at moderate concentrations, higher Cur concentrations (above 1 %) have been reported to reduce EB due to film discontinuities caused by Cur aggregation (Ma et al., 2017). Li et al. (2022) observed that EB declined from 13.22 % to 8.82 % as Cur content increased from 0 % to 3 %, before increasing again to 12.17 % at 7 % Cur. These trends highlight the importance of maintaining optimal Cur concentrations to ensure uniform dispersion and mechanical integrity.

The 0.5 % NC-Cur film demonstrated the best mechanical properties, with the highest TS, EB, and moderate elastic modulus (EM). These attributes are essential for food packaging applications, as mechanical properties influence both consumer acceptance and the ability to preserve food during storage and transportation (Salarbashi et al., 2021). The superior mechanical performance of the 0.5 % NC-Cur film makes it particularly suitable for packaging shrimp, where durability and flexibility are critical.

#### 3.1.5 X-ray diffraction (XRD)

**Fig. 3:**
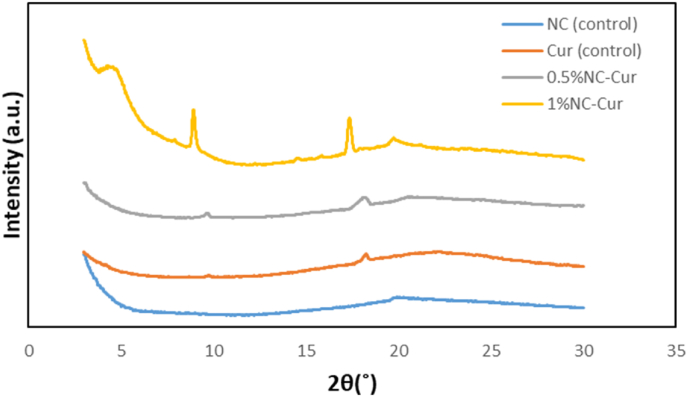
XRD patterns of NC (control), Cur (control), and 1 % NC-Cur intelligent food packaging films.

The X-ray diffraction (XRD) patterns of the Cur (control), NC (control), 0.5 % NC-Cur, and 1 % NC-Cur films are illustrated in Fig. 3. The primary diffraction peaks for the control samples were observed at 9.7058° for Cur and 19.9384° for NC. For the 0.5 % NC-Cur film, the peaks shifted slightly to 9.6788° and 20.9232°, while the 1 % NC-Cur film exhibited peaks at 9.2126° and 19.7353°. These shifts and the increased intensity and sharpness of the diffraction peaks in films containing NC indicate higher crystallinity compared to films without nanoclay.

The incorporation of NC into the biopolymer matrix generated prominent peaks near 20°, suggesting that NC was fully solubilised with sodium alginate (SA) and gelatin. These findings align with Jha (2021), who reported a diffraction peak at 20° in starch–chitosan–NC films, signifying complete solubilization of NC in the biopolymer matrix. Furthermore, the absence of the nanoclay peak at 7.21° (2θ) in all bio-nanocomposite films suggests successful intercalation of polymer chains between the nanoclay layers, consistent with observations by Jha (2020).

The enhanced dispersion of NC in the polymer matrix, as evidenced by the XRD patterns, is further supported by SEM images. The well-dispersed NC promotes nucleation, increasing the crystallinity of the films. This increased crystallinity correlates with improved mechanical properties, such as tensile strength, toughness, and resistance to moisture absorption, as higher crystallinity enhances the structural integrity of the films (Jha, 2020).

#### 3.1.6 Water contact angle (WCA)

The wettability and surface properties of food packaging materials are crucial for ensuring food safety, maintaining quality, extending shelf life, and preserving the appearance of packaged food. Wettability also plays an important role in intelligent packaging development by facilitating controlled interaction between food components and the packaging surface. The water contact angle (WCA) is a measure of the surface wettability of packaging materials, used to determine whether a surface is hydrophilic or hydrophobic (Etxabide et al., 2021a). A WCA of 90° is generally regarded as the threshold between hydrophilic and hydrophobic materials (Wang et al., 2022b).

The WCA values of the developed films are presented in Table 3. The 1 % NC-Cur film exhibited the highest WCA of 84.12 ± 3.14^c^ °, reflecting a 21.23 % increase in hydrophobicity compared to the Cur control film. The Cur control film showed the lowest hydrophobicity, with a WCA of 69.39 ± 1.75^a^ °. These results indicate that the hydrophobicity of the films increased with the inclusion of NC and the presence of Cur.

**Table 3:** Hydrophobic properties, barrier properties and solubility of food packaging films.

Films	Water Contact Angle (WCA)(°)	Water vapour permeability rate (WVPR) (g.m^2^.h^−1^) (72h)	Solubility ( %)	Moisture content ( %)
NC (control)	78.04 ± 2.47^b^	71.16 ± 0.22^b^	38.07 ± 1.46^d^	9.97 ± 0.76^a^
Cur (control)	69.39 ± 1.75^a^	72.20 ± 0.05^c^	61.84 ± 2.00^c^	26.69 ± 1.09^b^
0.5 %NC-Cur	80.52 ± 1.8^b,c^	72.72 ± 0.08^d^	22.50 ± 0.78^b^	23.93 ± 0.83^c^
1 %NC-Cur	84.12 ± 3.14^c^	66.27 ± 0.14^a^	16.61 ± 1.61^a^	17.55 ± 0.41^d^

∗The letters (a–d) indicate groups that are significantly different (p < 0.05).

The observed increase in hydrophobicity with Cur addition can be attributed to its inherent hydrophobic nature. The potential hydrogen-bonding interactions between Cur molecules and the biopolymer matrix likely reduced the exposure of hydrophilic groups on the film surface, thereby minimizing their interaction with water molecules (Etxabide et al., 2021a). Similarly, the increased concentration of NC contributed to surface hydrophobicity, potentially through improved dispersion within the matrix, which enhanced surface water repellence.

These findings highlight the role of Cur and NC in tailoring the surface properties of bio-based films, making them suitable for applications requiring improved moisture resistance, such as the packaging of perishable foods.

#### 3.1.7 Water vapour permeability

Water vapour permeability (WVP) is a critical parameter for evaluating the ability of packaging materials to maintain food safety and extend shelf life. Factors such as the hydrophobic/hydrophilic balance, crystallinity, pathway tortuosity, and structural defects influence film barrier properties (Fathi et al., 2022). Hydrophilic polymer films typically exhibit high WVP values, which limits their practical application in food packaging (Yildiz et al., 2021).

In this study, the inclusion of both curcumin (Cur) and nanoclay (NC) significantly improved the water vapour barrier properties of the films, as shown in Table 3. The 1 % NC-Cur film demonstrated the lowest water vapour permeability rate (WVPR) of 66.27 ± 0.14^a^ g·m^−2^·h^−1^, which represents an 8.95 % improvement compared to the Cur control film. The combination of Cur and NC yielded the optimal barrier properties, with increasing NC content further enhancing water resistance. Nanoclays enhance polymer barrier properties by creating a tortuous pathway within the polymer matrix, delaying the migration of water vapour molecules. The aspect ratio and dispersion of nanoclays throughout the polymer matrix are key factors influencing these barrier properties (Perera et al., 2024).

The presence of Cur also contributed to the reduced WVP of the films due to its hydrophobic nature. The benzene rings and long carbon chains in Cur introduce hydrophobic characteristics, forming tortuous routes that inhibit water vapour transmission. Additionally, the bulky benzene ring structures in Cur impede chain mobility, further limiting water vapour permeability. Similar results were reported in tara gum/polyvinyl alcohol-based nanofiber films, where Cur's hydrophobicity reduced WVP values (Yildiz et al., 2021).

Intermolecular interactions between Cur, NC, and the polymer matrix may also reduce the availability of hydrogen for hydrogen bonding with water molecules, decreasing the affinity of the films for water vapour. This phenomenon is supported by studies showing that a higher Cur concentration reduces WVP due to its hydrophobic properties (Ma et al., 2017; Liu et al., 2022). Furthermore, the compact structure created by smaller fiber diameters may lead to densely packed fibers, enhancing tortuous pathways and impeding water vapour movement (Yildiz et al., 2021).

The combined effects of Cur and nanoclay on reducing WVP are consistent with findings by Roy and Rhim (2020), who reported that adding Cur and ZnO to a carboxymethyl cellulose matrix significantly reduced WVP. Similarly, in the present study, the combination of NC and Cur effectively reduced the WVP of the films, making them suitable for applications requiring enhanced moisture resistance.

Minimizing WVP is essential to preserving food quality during storage (Taghinia et al., 2021). For shrimp packaging, a low WVP film is particularly advantageous, as it can significantly extend the shelf life of the product by limiting moisture transfer and spoilage.

#### 3.1.8 Water solubility and moisture content

Table 3 presents the water solubility and moisture content of the developed intelligent packaging films. The inclusion of nanoclay (NC) and curcumin (Cur) significantly reduced the water solubility of the control films. Among the four films, the Cur control film exhibited the highest solubility at 61.84 ± 2.0^c^ %. In contrast, the 1 % NC-Cur film demonstrated the lowest solubility, at 16.61 ± 1.61^a^ %, which is 3.72 times less soluble than the Cur control film. This reduction in water solubility highlights the beneficial effect of combining NC and Cur, as reduced solubility is a desirable characteristic for packaging materials.

Similar findings have been reported in previous studies. Li et al. (2022) observed that bacterial cellulose nanofiber-konjac glucomannan films exhibited high water solubility (63.44 %), but Cur-loaded films with 7 % Cur content reduced solubility to 28.6 7 %, demonstrating Cur's role in enhancing water resistance. Fathi et al. (2022) also reported that increasing Cur content (0–50 mg/g) led to a significant reduction in water solubility, attributed to the molecular interactions between Cur hydroxyl groups and the polymer matrix. These findings emphasize that hydrophobic additives like Cur inhibit the solubility of polysaccharide-based films, which are otherwise highly soluble due to their hydrophilic functional groups.

Water solubility is a critical property for packaging materials used with foods with high water content, as excessive solubility may cause films to absorb moisture and degrade, thereby limiting their application in food packaging (Mohseni-Shahri and Moeinpour, 2023). In this context, the reduced solubility observed in the NC-Cur films makes them more suitable for shrimp packaging, where durability in high-moisture environments is crucial.

Moisture content also plays a significant role in determining the barrier properties of packaging materials, as it directly influences their effectiveness in maintaining food quality. Table 3 shows that the NC control film exhibited the lowest moisture content (9.97 ± 0.76^a^ %), while the Cur control film displayed the highest moisture content, with a 2.67-fold increase compared to the NC control. The 1 % NC-Cur film had a moisture content that was 1.75 times higher than the NC control film. These results align with previous findings suggesting that nanoclay significantly improves moisture barrier properties by creating a tortuous path for water vapour, thereby reducing permeability (Rhim and Ng, 2007).

The addition of Cur further enhanced the barrier properties by lowering moisture permeability, consistent with the findings of Roy et al. (2022), who attributed this improvement to the hydrophobic nature of Cur. Hydrophobic compounds like Cur retain lower moisture levels, reducing water availability and thereby enhancing the film's ability to preserve food freshness and extend shelf life. For instance, Wang et al. (2022b) observed a moisture content of 21.29 % in 1 % w/v gelatin–1 % w/v chitosan–0.025 mmol/L Cur films, highlighting Cur's impact on moisture retention.

Minimizing water solubility and moisture content is essential for maintaining food quality during storage. Films with these properties act as effective moisture barriers, preventing spoilage and microbial growth caused by excess humidity (Bumbudsanpharoke and Ko, 2019). The 1 % NC-Cur film demonstrates superior water resistance and reduced solubility, making it an optimal choice for shrimp packaging applications, where maintaining freshness and extending shelf life are critical.

### 3.2 pH sensitivity studies

**Fig. 4:**
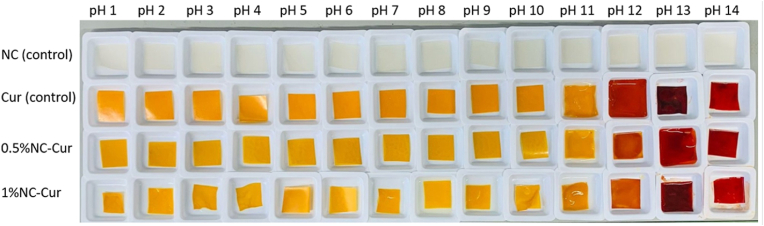
pH sensitive studies of intelligent food packaging materials NC (control), Cur (control), 0.5 % NC-Cur and 1 %NC-Cur against different pH solutions from a range of 1–14.

The pH sensitivity of the developed intelligent packaging films was evaluated to explore their potential as colorimetric indicators. Upon exposure to different pH buffers, the films displayed visible colour changes, with the colour shifting from yellow at neutral pH to orange and finally to red at alkaline pH (above pH 10), as shown in Fig. 4. The NC control film, which lacked curcumin (Cur), exhibited no colour change under varying pH conditions. This confirms that the colour variations in the films are attributable to the presence of Cur.

Cur's ability to undergo visible colour changes arise from its sensitivity to acid-base reactions. This property allows consumers to monitor food freshness by observing changes in packaging film colour without opening the package. Taghinia et al. (2021) reported that Cur-incorporated *Lallemantia iberica* seed gum films displayed a similar yellow-to-red transition when the pH changed from 5.7 to 9.9 during shrimp storage. Comparable colour shifts from yellow to orange and red were also observed in studies by Yildiz et al. (2021), Etxabide et al. (2021b), and Chen et al. (2020).

These pH-induced modifications in Cur are attributed to its structural transformations. Cur exists in equilibrium between its bis-keto and enol tautomeric forms, with the bis-keto form predominating under acidic pH (3–7). In acidic conditions, Cur acts as a hydrogen donor, leading to a π-π∗ electron excitation transition. At alkaline pH, the enolate form becomes dominant, and Cur acts as an electron donor. The phenolic hydroxyl group rapidly reacts with OH^−^ ions to generate phenoxide anions, which contribute to the observed red hue in alkaline conditions (Yildiz et al., 2021; Liu et al., 2018). This structural alteration underpins the suitability of Cur-based films for pH-sensitive intelligent packaging applications.

### 3.3 Antioxidant activity

The antioxidant activity of the intelligent packaging films was assessed using the DPPH radical scavenging method. Antioxidants are critical for protecting food from oxidative degradation, which can compromise flavour, nutritional value, and shelf life (Liu et al., 2022). The interaction of antioxidants with DPPH radicals decreases absorbance at 517 nm, indicating free radical scavenging activity (Ma et al., 2017).

**Fig. 5:**
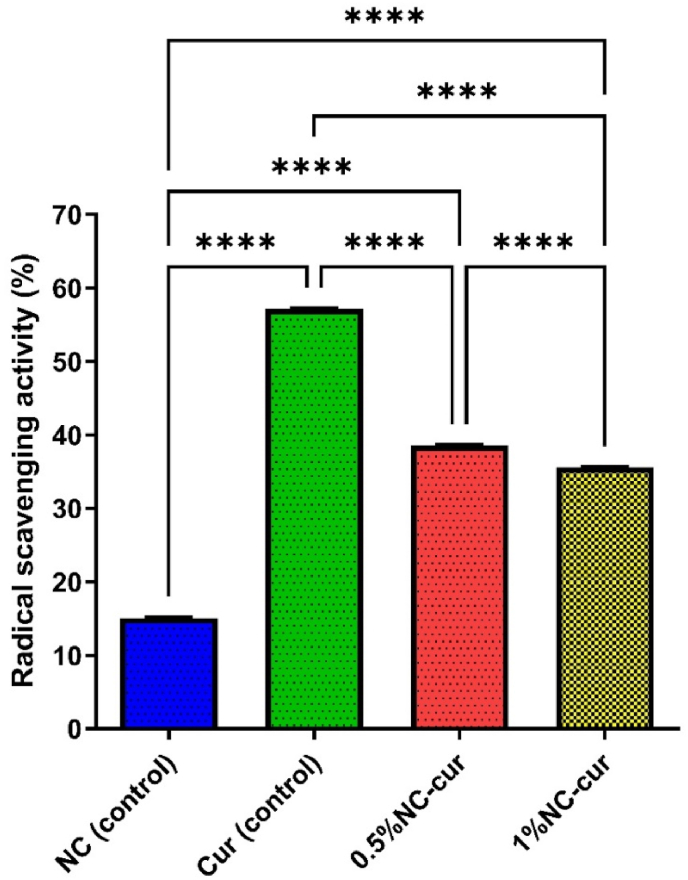
Antioxidant activity of NC (control), Cur (control), 0.5 %NC-Cur and 1 %NC-Cur intelligent food packaging films.

The antioxidant activity of the films is shown in Fig. 5. The Cur control film exhibited the highest antioxidant activity (57.14 ± 0.01^a^ %), which was 3.79 times greater than the NC control film (15.10 ± 0.05^d^ %). This is very similar to the previous studies were gelatin and SA is used. The DPPH radical scavenging capacity of fish gelatin films were 31.4 ± 2.5 %, which have been mainly attributed to the peptide fraction of gelatin (Kchaou et al., 2018). The gelatin-SA film showed around 5 % antioxidant activity (Dou et al., 2018).

Incorporating NC into the Cur film reduced its antioxidant activity to 35.55 ± 0.02^c^ % (1 % NC-Cur film), representing a 1.61-fold decrease compared to the Cur control film. This reduction is likely due to the small porous matrix created by NC, which limits the release of Cur. Despite this, the antioxidant activity of the 1 % NC-Cur film remained significantly higher than that of the NC control film, confirming that Cur is the primary contributor to the films’ antioxidant properties.

These findings align with the work of Roy and Rhim (2020), where the antioxidant activity of untreated carboxymethyl cellulose films increased from 1.9 % to 40.2 % with the addition of 1 wt % Cur. Similarly, Ma et al. (2017) reported that films without Cur exhibited low DPPH scavenging activity (1.81 %), which increased significantly upon Cur incorporation.

The antioxidant activity of Cur is largely attributed to its chemical structure, particularly the presence of phenolic hydroxyl and β-diketone groups, which donate hydrogen atoms to neutralize reactive oxygen species (ROS) (Liu et al., 2021). Cur's structure consists of two propenyl groups attached to a β-diketone (enol type) and benzene rings with phenolic hydroxyl and methoxy groups. The antioxidant properties arise from the ability of these groups to donate electrons or hydrogen atoms, effectively scavenging free radicals.

The 1 % NC-Cur film offers a balance between antioxidant activity and barrier properties, making it suitable for protecting oxidation-sensitive foods, such as fish and shrimp. The incorporation of Cur in packaging films enhances their antioxidant potential and contributes to food preservation by reducing oxidative spoilage and extending shelf life (Taghinia et al., 2021).

### 3.4 Antimicrobial activity

Antimicrobial properties are a critical feature of food packaging films, as foodborne pathogens can compromise food quality, cause spoilage, and pose health risks (Perera et al., 2022a, Perera et al., 2022b). The antimicrobial activity of the developed intelligent packaging films was assessed over 0 and 24 h against two foodborne pathogens: *Escherichia coli* (Gram-negative) and *Staphylococcus aureus* (Gram-positive).

The Cur control film exhibited complete inhibition against both pathogens, outperforming the NC control film. For *E. coli*, log reductions of 1.89 ± 0.12 and 0.74 ± 0.07 CFU/mL were observed for the 0.5 % NC-Cur and 1 % NC-Cur films, respectively, compared to the NC control. For *S. aureus*, log reductions of 1.11 ± 0.04 and 0.03 ± 0.01 CFU/mL were recorded for the 0.5 % NC-Cur and 1 % NC-Cur films, respectively, when compared to the NC control. These results indicate that the addition of Cur significantly enhanced the antimicrobial activity of the packaging films, with 0.5 % NC-Cur showing superior performance compared to 1 % NC-Cur.

The antimicrobial efficacy of Cur can be attributed to its ability to disrupt microbial cell membranes. Curcumin interferes with cellular processes by causing the release of cytoplasmic contents, organelle disruption, and enzyme inhibition. These actions result in the leakage of proteins, nucleic acids, and other cellular components, ultimately leading to microbial cell death (Liu et al., 2021).

While NC itself lacks intrinsic antimicrobial properties, its incorporation into the film matrix enhances the controlled release of Cur. Active molecules intercalated into NC layers are gradually released into the environment, prolonging the antimicrobial effect. However, studies have shown that increasing NC concentration reduces Cur release, which may explain the lower antimicrobial activity observed in 1 % NC-Cur films compared to 0.5 % NC-Cur (Perera et al., 2024).

Additionally, NC improves the moisture barrier properties of packaging films, which may indirectly inhibit microbial growth by maintaining lower moisture content (Smith et al., 2019). This study confirms that NC aids in controlling moisture levels, creating an environment less conducive to microbial proliferation. However, variations in results may arise due to the active role of Cur within the matrix.

Although the present study observed limited antimicrobial activity over 24 h, the controlled release of Cur from NC suggests that the films can provide prolonged antimicrobial protection. The synergistic action of NC and Cur offers a promising solution for enhancing food safety in active packaging applications.

### 3.5 Migration of curcumin

**Fig. 6:**
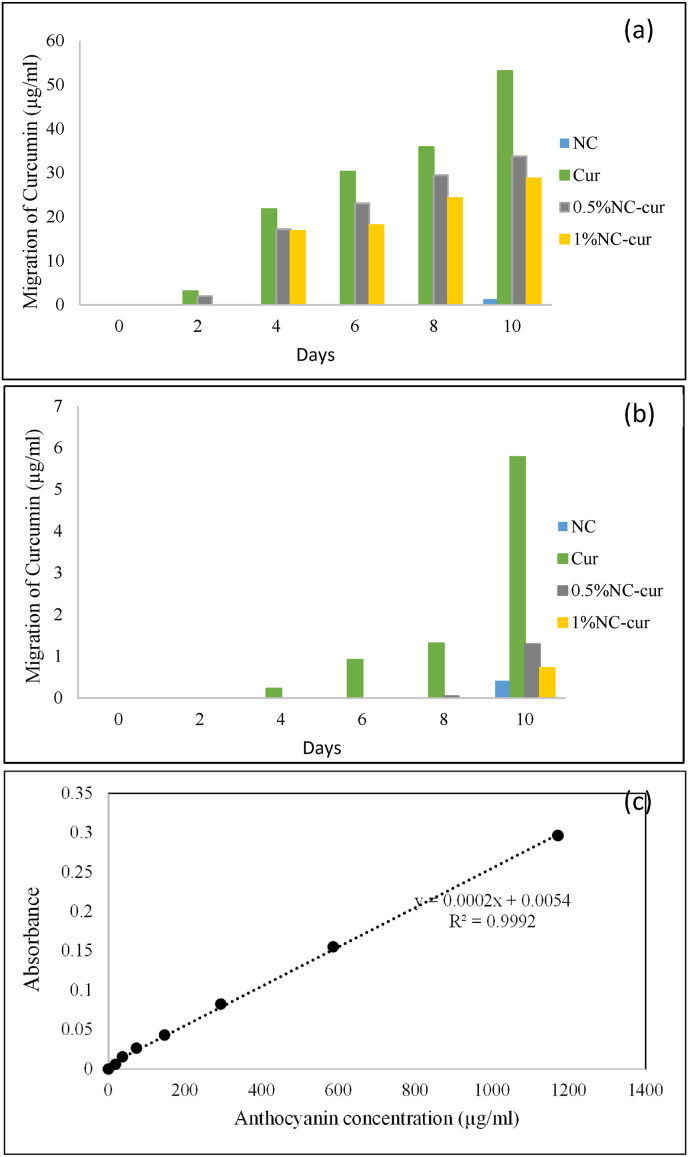
Migration of Cur into food stimulants (a) Olive oil and (b) 3 % acetic acid in NC (control), Cur (control), 0.5 % NC-Cur and 1 %NC-Cur food packaging materials during a time period of 10 days and (c) the standard curve of Cur.

The migration of curcumin (Cur) was evaluated in two food simulants: olive oil (stimulant D) and 3 % (v/v) aqueous acetic acid (stimulant B), as shown in Fig. 6. The level of migration was quantified using a Cur standard curve. The highest migration level was observed in olive oil for the Cur control film, reflecting Cur's lipophilic nature. As the concentration of nanoclay (NC) increased, the migration of Cur decreased, with the 1 % NC-Cur film showing the lowest migration level of 28.76 ± 0.02^c^ μg/mL, which is 1.84 times lower than the Cur control film.

In 3 % (v/v) aqueous acetic acid, significantly lower Cur migration levels were observed across all films. The Cur control film exhibited the highest migration at 5.79 ± 0.013^a^ μg/mL, while the 1 % NC-Cur film showed a migration level of 0.72 ± 0.002^c^ μg/mL, an 8.04-fold reduction compared to the Cur control.

A strong positive correlation was observed between antioxidant activity and Cur migration levels in both food simulants: olive oil (*r* = 0.99) and 95 % ethanol (*r* = 0.86). These findings suggest that DPPH radical scavenging activity depends on the level of Cur migration.

According to the European Plastics Regulation (EU) No. 10/2011, the cumulative migration limit for all substances in food contact materials is set at 60 mg/kg (equivalent to 60 μg/mL or 0.06 mg/mL in appropriate units) (European Commission, 2019). The migration levels of Cur in all tested food simulants were below this threshold, confirming compliance with EU regulations. The incorporation of NC into the food packaging films reduced Cur migration, indicating that NC effectively limits the release of active compounds into food products.

If Cur does migrate into the packaged food, such as shrimp in this study, it remains within the EU-specified migration limits and provides added benefits due to its antioxidant and antimicrobial properties. Cur is widely recognized as a safe, medicinal herb with bioactive properties, and its migration could enhance the functionality of the packaged food by improving its antioxidant capacity (El-Saadony et al., 2023).

### 3.6 Biodegradability studies of the intelligent food packaging films

**Fig. 7:**
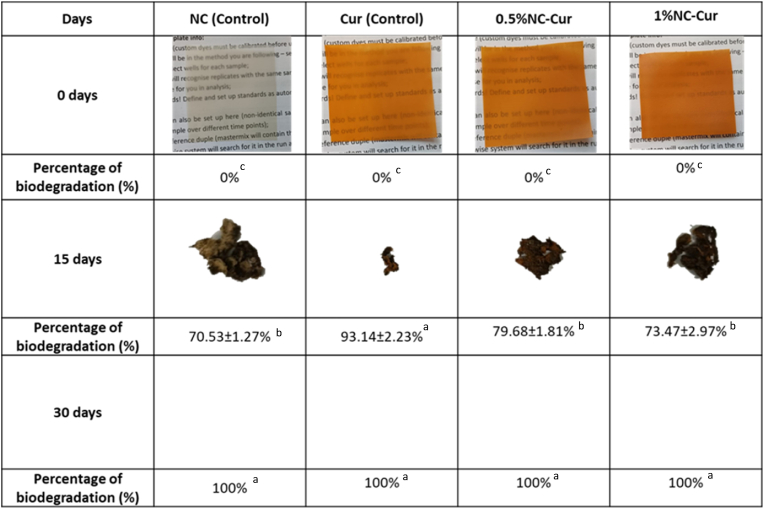
Biodegradation of NC (control), Cur (control), 0.5 %NC-Cur and 1 %NC-Cur food packaging films for a time period of 30 days.

Biodegradability studies are essential for assessing the environmental impact of food packaging materials. In this investigation, all films degraded completely within 30 days, as shown in Fig. 7. The presence of nanoclay (NC) influenced the rate of biodegradation, while curcumin (Cur) had no significant effect.

After 15 days, the NC control film exhibited the lowest biodegradation rate (70.53 ± 1.27^b^ %), likely due to the incorporation of NC. The reduced biodegradation rate can be attributed to the structural characteristics of NC, including its ability to form strong hydrogen bonds with the biopolymer matrix. This interaction reduces the polymer's sensitivity to water and enhances cohesion within the matrix, slowing down the biodegradation process.

Similar findings were reported by Yeasmin et al. (2020), who studied the biodegradation of pullulan, TEMPO cellulose nanofibrils, and montmorillonite (MMT)-based food packaging materials using the soil burial method. Their results demonstrated that the addition of MMT nanoclay decreased the biodegradation rate due to strong hydrogen bonding between the nanoclay's hydroxyl groups and the pullulan matrix. This increased the cohesion of the polymer and reduced its water sensitivity, thereby slowing degradation.

While NC delayed biodegradation during the initial 15 days, all films, regardless of NC concentration, achieved complete biodegradation by 30 days. This demonstrates that the developed films are environmentally friendly and suitable for sustainable packaging applications.

### 3.7 Application of films for indicating the freshness of shrimp

**Fig. 8:**
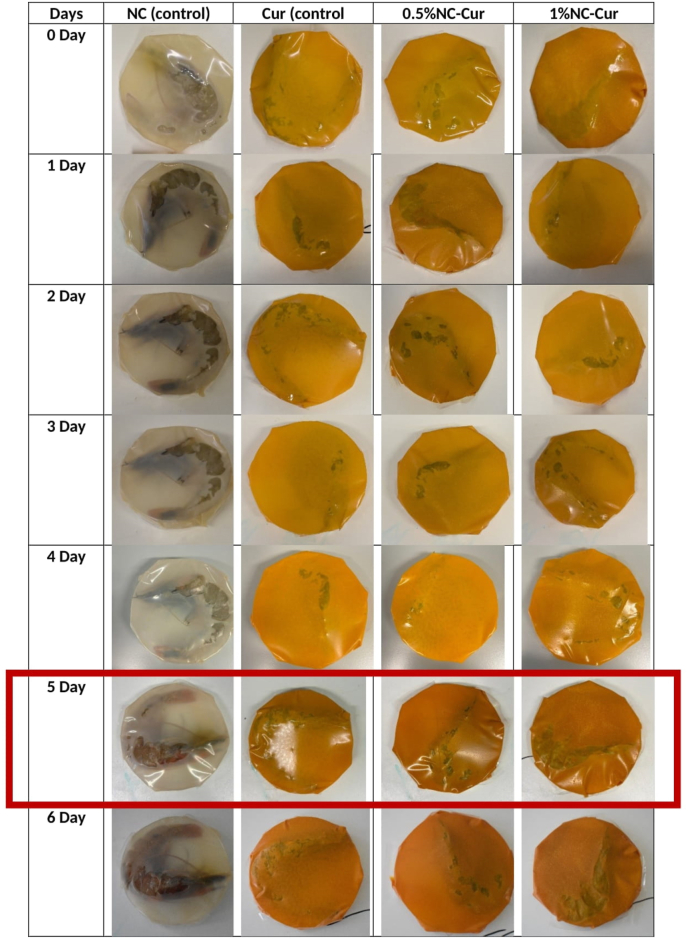
Application study of shrimp with food packaging materials packaged with NC (control), Cur (control), 0.5 % NC-Cur, and 1 % NC-Cur during a 14-day time period at 4 °C.

**Fig. 9:**
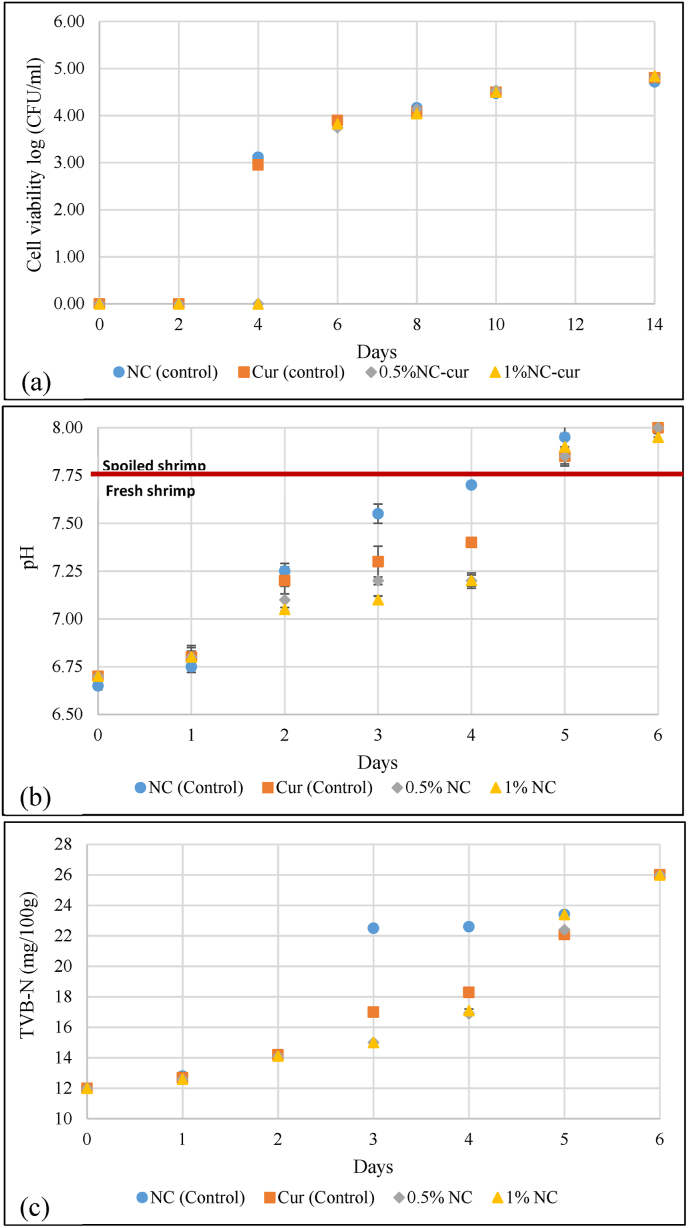
Changes in shrimp when packaged in NC (control), Cur (control), 0.5 %NC-Cur and 1 %NC-Cur (a) total bacterial count, (b) pH changes and (c) TVB-N levels.

Shrimp were used as a model to evaluate the applicability of intelligent packaging films for real-time monitoring and maintaining the freshness of protein-rich foods. The films’ colour changed from yellow to orange by the fifth day of storage at 4 °C, except for the NC control films, which exhibited no colour change as depicted in Fig. 8. This observation suggests that shrimp spoilage begins after the fourth day of storage. Similar results were observed in the study by Taghinia et al. (2021), where the packaging membrane changed from yellow to orange after four days. Shrimp spoilage was confirmed by evaluating pH, total volatile basic nitrogen (TVB-N), total bacterial growth, and changes in film colour during six days of storage as shown in Fig. 9.

The pH of fresh shrimp was approximately 6.7 ± 0.1^a^ at the start of the experiment and steadily increased to 7.99 ± 0.02^b^ by the sixth day. Shrimp are considered unsuitable for consumption when their pH exceeds 7.5. The NC control film surpassed this threshold by the third day, with a pH of 7.53 ± 0.02^b^. In contrast, shrimp wrapped with Cur control films and NC-Cur films remained below the spoilage threshold until day five. Shrimp packaged in 0.5 % NC-Cur and 1 % NC-Cur films had pH values of 7.48 ± 0.01^c^ and 7.47 ± 0.01^c^, respectively, on day five, demonstrating a delayed spoilage process. These findings are consistent with the results of Kuswandi (2012), who reported that Cur-based sensors reacted to increasing volatile base concentrations in shrimp, with membrane colour changes correlating to microbial activity.

TVB-N, a spoilage marker produced during microbial decomposition of proteins, increased as storage time progressed. High concentrations of TVB-N, including ammonia, dimethylammonium, and trimethylamine, are known to indicate shrimp spoilage, particularly when exceeding 20 mg/100 g. A correlation between TVB-N levels and pH changes was observed, with microbial activity being a key contributor to both. Shrimp wrapped in NC-Cur films showed no visible microbial growth up to six days, while shrimp wrapped in NC and Cur control films exhibited visible growth after four days. These results indicate that the combined effect of NC and Cur delayed spoilage by extending shrimp shelf life by two days. This delay is likely due to the controlled release of Cur from the NC-Cur films, as observed in migration studies.

The films’ visual colour changes (as shown in Table 4) correlated with spoilage indicators such as pH and TVB-N. The reduction in lightness (*L*), increase in redness (*a*), and decrease in yellowness (*b*) observed over time confirmed the transition from yellow to orange as spoilage progressed. These results align with findings by Wang et al. (2022a) and Yildiz et al. (2021), who reported similar trends in films used to package shrimp and chicken. The increase in *a* value and decrease in *b* values were attributed to an alkaline environment caused by TVB-N accumulation.

**Table 4:** Changes of the film colour during shrimp storage; L∗(lightness), a∗ (redness) and b∗ (yellowness).

	L∗ (Lightness)				a∗ (Red-Green)				b∗ (Yellow-Blue)			
Days	NC(Control)	Cur (Control)	0.5 %NC-Cur	1 %NC-Cur	NC(Control)	Cur (Control)	0.5 %NC-Cur	1 %NC-Cur	NC(Control)	Cur (Control)	0.5 %NC-Cur	1 %NC-Cur
0	84.21 ± 1.46^a^	67.15 ± 0.28^b^	64.21 ± 0.37^c^	65.72 ± 0.31^b^	(−)0.17 ± 0.05^d^	29.54 ± 0.03^a^	28.82 ± 0.29^a^	26.99 ± 0.36^b^	10.61 ± 0.24^a^	64.09 ± 0.34^b^	59.77 ± 0.77^c^	61.8 ± 0.37^b^
2	84.486 ± 0.75^a^	54.54 ± 0.23^b^	45.93 ± 0.13^d^	49.88 ± 0.18^c^	1.43 ± 0.04^d^	31.3 ± 0.30^c^	38.24 ± 0.06^a^	38.44 ± 0.09^a^	4.90 ± 0.06^b^	53.87 ± 0.37^a^	53.60 ± 0.15^a^	57.06 ± 0.1^a^
4	80.26 ± 0.11^a^	48.47 ± 0.15^c^	45.75 ± 0.04^c^	42.64 ± 0.23^d^	1.24 ± 0.79^d^	32.20 ± 0.09^c^	34.48 ± 0.07^b^	34.38 ± 0.11^b^	3.86 ± 0.11^b^	43.87 ± 0.22^a^	33.60 ± 0.04^b^	37.06 ± 0.22^b^
6	85.19 ± 0.17^a^	54.56 ± 0.31^b^	49.22 ± 0.28^c^	44.03 ± 0.42^d^	1.42 ± 0.09^c^	41.48 ± 0.27^b^	41.20 ± 0.23^b^	42.44 ± 0.23^a^	4.97 ± 0.13^b^	44.38 ± 0.59^a^	37.96 ± 0.43^b^	36.23 ± 0.87^b^
8	83.49 ± 1.55^a^	44.22 ± 0.60^b^	43.15 ± 0.48^b^	36.62 ± 1.61^c^	0.38 ± 0.29^c^	47.59 ± 0.79^b^	43.66 ± 0.08^c^	48.09 ± 0.48^a^	9.75 ± 0.10^a^	37.21 ± 0.06^b^	27.97 ± 0.33^c^	33.98 ± 0.89^b^
10	80.75 ± 0.55^a^	45.88 ± 0.51^b^	37.32 ± 0.43^c^	40.02 ± 0.06^c^	2.27 ± 0.16^c^	43.23 ± 0.06^c^	44.63 ± 0.39^b^	47.70 ± 0.49^a^	5.54 ± 0.14^b^	28.67 ± 0.25^c^	26.63 ± 0.54^c^	29.12 ± 0.4^b^
14	78.92 ± 0.31^a^	50.26 ± 0.24^b^	41.77 ± 0.47^c^	40.43 ± 0.15^c^	0.63 ± 0.02^c^	45.72 ± 0.27^b^	49.38 ± 0.85^a^	48.43 ± 0.07^a^	10.4 ± 0.12^a^	24.48 ± 0.50^b^	22.75 ± 0.19^c^	29.19 ± 0.08^b^

The application of intelligent packaging films offers a practical approach to real-time monitoring of shrimp freshness. The films’ visible colour changes provide a clear indication of spoilage, allowing consumers to identify unfit food without opening the package. The findings are consistent with Mohammadalinejhad et al. (2020), who reported a strong correlation between TVB-N levels, pH, and ΔE in anthocyanin-based packaging films. While dark brick-red colours were observed during pH sensitivity tests, such colours were not detected in the shrimp packaging study, as the pH of spoiled shrimp did not exceed 13.

This study demonstrates that intelligent films effectively monitor and indicate shrimp spoilage through visible colour changes, correlating with microbial activity, TVB-N accumulation, and pH variations. These films provide a valuable tool for ensuring food safety and reducing waste in the storage and distribution of perishable protein-rich foods.

## 4 Conclusion and future perspectives

The developed intelligent food packaging films, composed of curcumin (Cur), gelatin, sodium alginate (SA), and nanoclay (NC), demonstrate strong potential for shrimp packaging. The incorporation of Cur enhanced antioxidant activity by 35.55-fold and improved pH sensitivity, mechanical strength, and water solubility (3.72-fold lower than the control). NC further enhanced the films' barrier properties and hydrophobicity and controlled the release of Cur, minimizing migration while maintaining biodegradability within 30 days. The films monitored shrimp spoilage through pH sensitivity, with visible colour changes corresponding to microbial growth and spoilage indicators. Antioxidant properties contributed to maintaining shrimp freshness by delaying oxidative spoilage, while the controlled release of Cur extended shrimp shelf life by an additional two days. By combining Cur's antioxidant and pH-sensing capabilities with NC's controlled release functionality, these films offer a sustainable solution for improving food quality, reducing waste, and enhancing the safety of protein-rich foods.

## CRediT authorship contribution statement

**Kalpani Y. Perera:** Conceptualization, Formal analysis, Data curation, Investigation, Methodology, Laboratory work, Writing – original draft, Writing – review & editing. **Sneha Sabu Mathew:** Investigation, Methodology. **Luana de S.C. Carnaval:** Investigation, Methodology. **Dileswar Pradhan:** Investigation, Methodology. **Amit K. Jaiswal:** Funding acquisition, Project administration, Conceptualization, Resources, Supervision, Writing – review & editing, All authors have read and agreed to the published version of the manuscript. **Swarna Jaiswal:** Conceptualization, Formal analysis, Validation, Resources, Supervision, Writing – review & editing.

## Declaration of competing interest

The authors declare that they have no known competing financial interests or personal relationships that could have appeared to influence the work reported in this paper.

## Data Availability

Data will be made available on request.
